# A New Approach for On-Demand Generation of Various Oxygen Tensions for *In Vitro* Hypoxia Models

**DOI:** 10.1371/journal.pone.0155921

**Published:** 2016-05-24

**Authors:** Chunyan Li, Wayne Chaung, Cameron Mozayan, Ranjeev Chabra, Ping Wang, Raj K. Narayan

**Affiliations:** 1 Cushing Neuromonitoring Laboratory, The Feinstein Institute for Medical Research, Manhasset, New York, United States of America; 2 Department of Neurosurgery, Hofstra Northwell School of Medicine, Manhasset, New York, United States of America; 3 Center for Translational Research, The Feinstein Institute for Medical Research, Manhasset, New York, United States of America; Michigan Technological University, UNITED STATES

## Abstract

The development of *in vitro* disease models closely mimicking the functions of human disease has captured increasing attention in recent years. Oxygen tensions and gradients play essential roles in modulating biological systems in both physiologic and pathologic events. Thus, controlling oxygen tension is critical for mimicking physiologically relevant *in vivo* environments for cell, tissue and organ research. We present a new approach for on-demand generation of various oxygen tensions for *in vitro* hypoxia models. Proof-of-concept prototypes have been developed for conventional cell culture microplate by immobilizing a novel oxygen-consuming biomaterial on the 3D-printed insert. For the first time, rapid (~3.8 minutes to reach 0.5% O_2_ from 20.9% O_2_) and precisely controlled oxygen tensions/gradients (2.68 mmHg per 50 μm distance) were generated by exposing the biocompatible biomaterial to the different depth of cell culture media. In addition, changing the position of 3D-printed inserts with immobilized biomaterials relative to the cultured cells resulted in controllable and rapid changes in oxygen tensions (<130 seconds). Compared to the current technologies, our approach allows enhanced spatiotemporal resolution and accuracy of the oxygen tensions. Additionally, it does not interfere with the testing environment while maintaining ease of use. The elegance of oxygen tension manipulation introduced by our new approach will drastically improve control and lower the technological barrier of entry for hypoxia studies. Since the biomaterials can be immobilized in any devices, including microfluidic devices and 3D-printed tissues or organs, it will serve as the basis for a new generation of experimental models previously impossible or very difficult to implement.

## Introduction

The development of *in vitro* disease models that can closely mimic the functions of human disease has captured increasing attention in recent years [1–3]. By individually manipulating mechanical, chemical and cellular components, it becomes possible to overcome the major limitations of whole-animal models which have limited relevance to humans and are difficult to identify or vary critical cellular and molecular contributors to disease. Oxygen tensions and gradients play essential roles in modulating biological systems in both physiologic and pathologic events [4, 5]. Oxygen tension impacts a variety of vital biological processes including but not limited to embryonic development, metabolism, and angiogenesis. Hypoxia, a condition of low oxygen tension, is a major factor in the pathophysiology of many acute and chronic diseases [6, 7]. Hypoxia is involved in the development of pathological conditions including systemic inflammatory response, tumorigenesis, and cardiovascular disease such as ischemic heart disease and pulmonary hypertension. Controlling oxygen tensions or gradients is thus critical for mimicking physiologically relevant *in vivo* environments for cell, tissue and organ research.

Many technologies and devices have been developed for controlling oxygen in both macro and micro environments. Current techniques for global oxygen control of macro-environments include hypoxic chambers/workstations/perfusion chambers or directly adding oxygen scavenging agents. Critical challenges remain for these approaches including only providing a single condition of a hypoxic level at a time, slow equilibration time [8] and being not able to provide physiological oxygen gradients that are found *in vivo* or control at the microscale. Moreover, oxygen scavenging agents alter the cell growth environment and may affect cellular responses [9]. To fulfill this unmet need, microfluidic approaches have been developed to overcome the inherent limits of these current approaches [10–12]. They allow for multiple hypoxic conditions, rapid equilibrium time, and the generation of oxygen gradients [13–20]. While they better mimic the architecture and microenvironment of living tissue, their operation requires bulky flow control instrument and unwieldy interconnections that limit their practical usage in the laboratory setting.

In this study, we developed a new approach for on-demand generation of various oxygen tensions for *in vitro* hypoxia models to overcome these challenges. Proof-of-concept prototypes have been developed for conventional cell culture microplates (96-well and 24-well cell culture microplates) by immobilizing a novel oxygen-consuming biomaterial on the 3D-printed insert. First, the effects of different biomaterial compositions were investigated. Oxygen tensions at different distance from the optimized biomaterial and the response time to reach each steady state oxygen tension were then measured. The dynamic modulation of oxygen tensions was further evaluated for the ischemia-reperfusion injury model. After that, the cytotoxicity tests of the biomaterials were performed. Finally, the developed prototypes were evaluated by examining the release of hypoxia-inducible factor 1α (HIF-1α) and tumor necrosis factor α (TNF-α) by macrophages under different hypoxic levels.

## Materials and Methods

### Biomaterials

The biomaterial is a hydrogel comprising glucose oxidase (G7141, Sigma) and catalase (C1345, Sigma) enzymes cross-linked with chitosan (1% (w/v) chitosan acetic acid solution; 448877, Sigma) and glutaraldehyde (0.125%; G4004, Sigma). Glucose oxidase density and the ratio of glucose oxidase to catalase were adjusted from 0 U/cm^2^ to 15 U/cm^2^ and from 1:0 to 1:50, respectively. The glucose oxidase and catalase enzymes were diluted with distilled water to have the density of 25 U/μl and 625 U/μl, respectively. A 1% chitosan solution was prepared by dissolving 1.0 g of chitosan in 100 ml of 1.0% acetic acid. For the biomaterial with 0 U/cm^2^, 10 μl glutaraldehyde solution (1.25%) was added into the 100 μl 1% chitosan solution. For the biomaterial with glucose density from 1.5 U/cm^2^ to15 U/cm^2^, 1–10 μl glucose oxidase solution and 2–20 μl catalase solution were dissolved in 100 μl 1% chitosan solution, and then 10 μl glutaraldehyde solution (1.25%) was added. The biomaterials were coated on the targeted surface and allowed to cure at 4°C for 24 hours prior to use.

Glucose oxidase (GOX) enzyme oxidizes D-glucose molecule, which is one of major components of the cell culture medium (most cell culture media have glucose concentrations between 5 and 25 mM), into gluconic acid and hydrogen peroxide. Catalase (CAT) enzyme decomposes hydrogen peroxide into water and oxygen. Based on the following chemical equations, two oxygen molecules are consumed and only one is produced, resulting in a net loss of oxygen [21].

$$\text{Glucose}+{\text{O}}_{2}+{\text{H}}_{2}\text{O}\overset{\text{GOX}}{\to }\text{Gluconic Acid}+{\text{H}}_{2}{\text{O}}_{2}$$

$${\text{H}}_{2}{\text{O}}_{2}\overset{CAT}{\to }{\text{H}}_{2}\text{O}+\frac{1}{2}{\text{O}}_{2}$$

Net reaction:

$$\text{Glucose}+{\frac{1}{2}\text{O}}_{2}\overset{\text{GOX}+\text{CAT}}{\to }\text{Gluconic Acid}$$

### Design and fabrication of the insert for cell culture microplate

**Fig 1:**
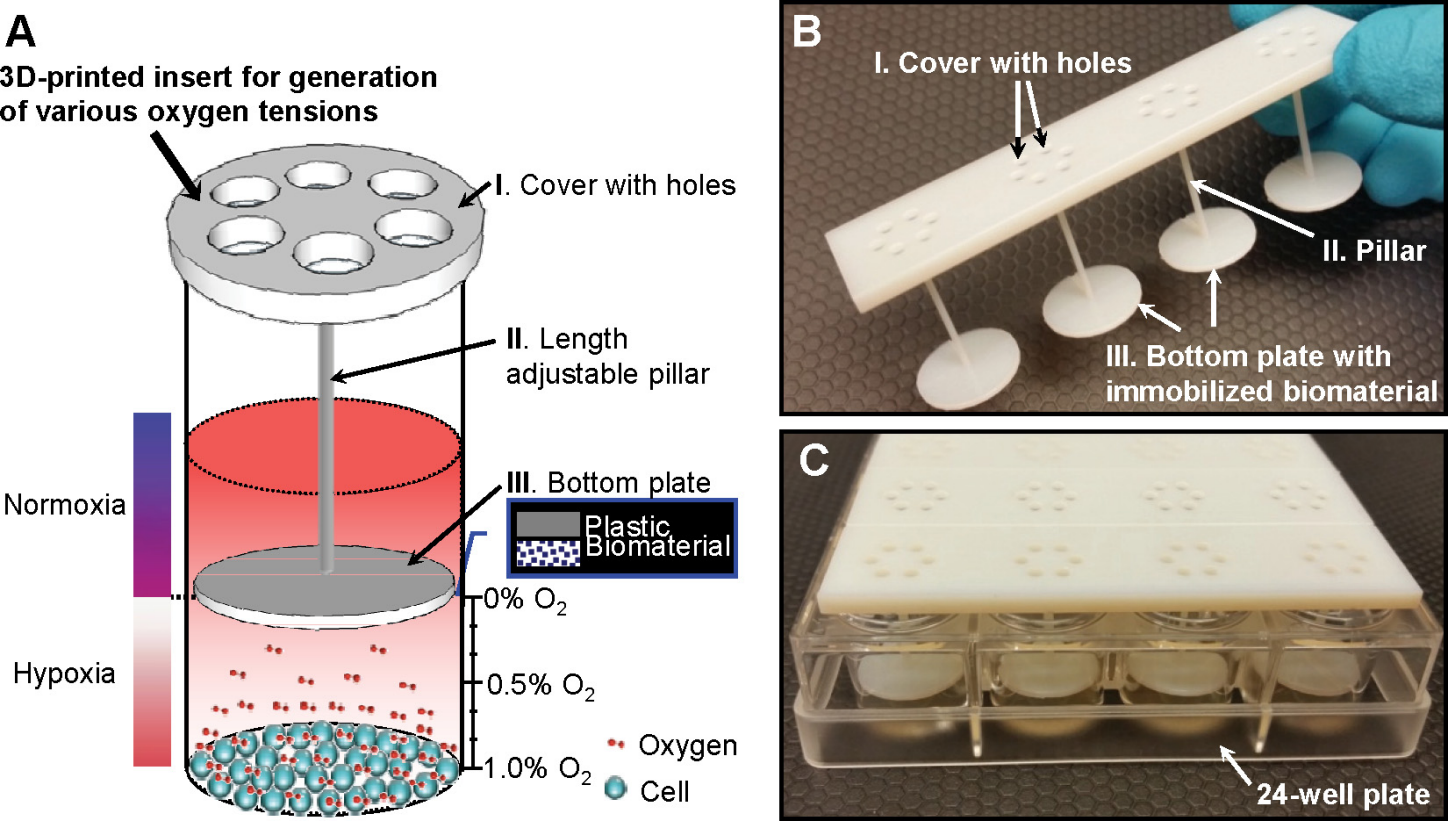
A new approach for on-demand generation of various oxygen tensions. (A) Conceptual illustration of the 3D-printed insert with immobilized biomaterial for on-demand generation of various oxygen tensions for *in vitro* cell cultures. The biomaterial consisting of glucose oxidase and catalase enzymes consumes oxygen in the cell culture media without interfering with the testing environment. (B) Photo of a 3D-printed insert for 24-well microplate. It can control oxygen tension in one row of 4 wells. (C) Multiple inserts with different pillar lengths were placed in the 24-well microplate to generate various oxygen tensions at the same time.

The insert device was designed to integrate with the multiwell cell culture microplates and fabricated through 3D printing technology. Fig 1A shows the conceptual illustration of the insert with immobilized biomaterial for generation of various oxygen tensions. The insert consists of 3 parts, cover, pillar and bottom plate. The cover (thickness: 2 mm) is designed to be put on the top of each microplate well. It allows keeping the fixed distance from the bottom plate. Each cover has 6 holes (diameter: 2 mm) for oxygen and carbon dioxide gases exchanging in cell culture incubator. During incubation, 1 mm thick PDMS membrane (Sylgard 184) is attached on the cover to prevent moisture evaporation while allowing gas exchanges. The pillar (diameter: 1.5 mm) has various length which controls the distance between the cultured cells and bottom plate. The biomaterial is immobilized on the bottom plate surface toward the cultured cells (thickness: 1.5 mm). The diameter of the bottom plate is 6 mm and 15.2 mm for 96-well and 24-well microplates, respectively. The pillars extend into each well leaving various gaps between the biomaterial and the cell culture. Since oxygen consuming biomaterial is immobilized in the chitosan matrix, oxygen consumption only occurs on the surface of biomaterial. The lowest oxygen tension is achieved on the biomaterial surface, and increases with radial distance from the biomaterial surface. The absolute oxygen tension is determined by 20.9% ambient atmospheric oxygen diffusion through microplate (polystyrene) and oxygen consumption by biomaterials. As a result, precisely-controlled oxygen tensions are achieved at the different distances from the biomaterial. Accordingly, the oxygen gradient which forms radial and axial diffusion of oxygen from the biomaterial is generated.

The insert was designed using CAD tools, and ordered by mail [22]. The developed 3D-printed insert for 24-well cell culture microplate is shown in Fig 1B and 1C. It was designed to control oxygen tension in one row of 4 wells each. Up to 6 inserts generating as much as 6 difference oxygen tensions can be inserted in the 24-well cell culture microplate at the same time. The insert with various pillar lengths can produce different oxygen tensions.

### Measurement of oxygen tension

Oxygen tensions were measured using a precision temperature compensated fiber-optic oxygen measurement system (OXYMICRO-Fiber-optic sensor; World Precision Instruments). A 21 gauge hole was drilled on the bottom plate in order to insert the oxygen sensing probe. The tip of the oxygen microsensor was aligned to the bottom surface of the 96-well plate. The 3D-printed insert for 96-well was repositioned using a precision XYZ manipulator to control the distance between the biomaterial and oxygen microsensor.

### Cell culture

All experiments were performed in accordance with the recommendations in the Guide for the Care and Use of Laboratory Animals of the National Institutes of Health and the protocol was approved by the Institutional Animal Care and Use Committee (IACUC) at the Feinstein Institute for Medical Research. Male Sprague-Dawley (SD) rats (250–350 g) purchased from Charles River Laboratories (Wilmington, MA) were used. Rats were housed in a temperature-controlled room on a 12-h light/dark cycle and fed a standard Purina rat chow diet. At the day of harvesting peritoneal macrophages, rats were anesthetized by isoflurane inhalation, and the abdomen was shaved and washed with 10% povidone iodine. One hundred ml of HBSS were injected to the peritoneal cavity and the rats were gently shaken for 2 min. A small incision on the abdomen was performed to insert a 20-gauge needle to collect all the peritoneal fluid. Then, the rats were rapidly and painlessly euthanized using carbon dioxide exposure followed by exsanguination, as recommended by the American Veterinary Medical Association (AVMA) Guidelines for the Euthanasia of Animals: 2013 Editions. All surgery was performed under isoflurane anesthesia, and all efforts were made to minimize suffering.

Extracted peritoneal macrophages were cultured at 1 X 10^6^ cells/ml RPMI 1640 media in 96-well and 24-well plate [23]. The plates were incubated overnight at 37°C in a humidified, 5% CO_2_ incubator. After replacing the overnight media with fresh one, the sterilized inserts that can generate various oxygen tensions were placed into the pate.

### Biochemistry and ELISA assay analysis

Hydrogen peroxide assay kit (Catalog# STA-344, Cell Biolabs Inc.) and glucose (GO) assay kit (Catalog# GACO20-1KT, Sigma Aldrich Inc.) were used to measure the levels of hydrogen peroxide and glucose. MTS viability assay (Catalog# G3580, Promega) for mammalian cells was performed to test the biocompatibility of the biomaterials. TNF-α ELISA assay set (Catalog# 558535, BD) was used to evaluate the effect of hypoxia on peritoneal macrophages.

### HIF-1α western blotting

The levels of HIF-1α protein from hypoxia induced peritoneal macrophage cells were determined by western blot analysis using HIF-1 alpha antibody (AF1935, R&D). The β-actin western blot results from each individual line were used as the internal controls. The band densities from each line were digitalized using Bio-Rad densitometry image system. The ratio of HIF-1α / β-actin from each group were calculated, averaged and graphed in the histogram. The control group (cells only) and matrix group (no enzymes) were included as the background controls.

### Statistical analysis

All data were expressed as mean ± SD (n = 4–12) and compared by one-way ANOVA and the Student Newman-Keuls methods. P<0.05 was considered statistically significant.

## Results

### Effect of glucose oxidase to catalase ratio in biomaterial

**Fig 2:**
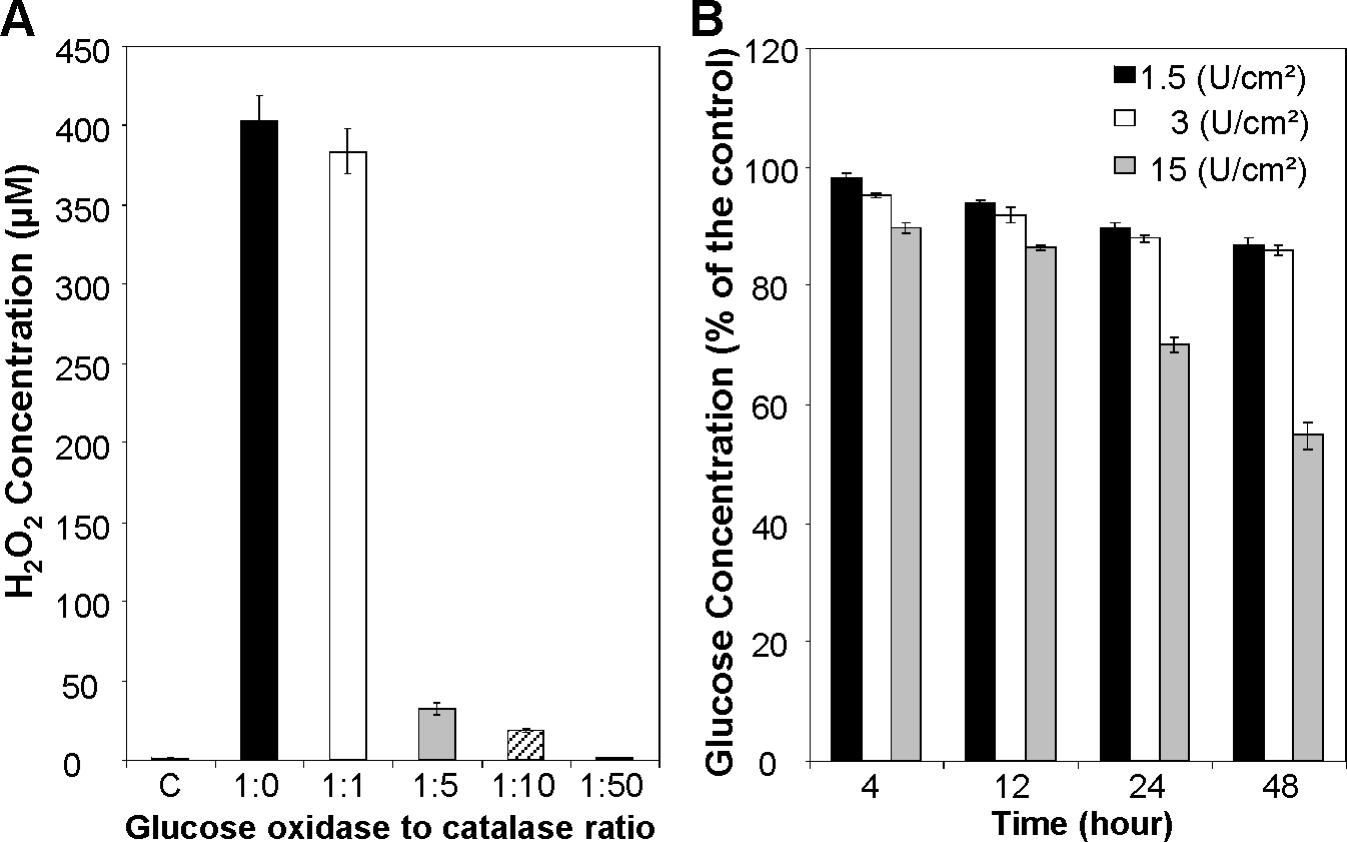
Effect of glucose oxidase to catalase ratio in biomaterial. (A) Hydrogen peroxide concentration after 24 hours incubation. The ratio of glucose oxidase to catalase was adjusted to 1:0, 1:1, 1:5, 1:10 and 1:50. (B) D-glucose concentration after 4, 12, 24 and 48 hours incubation. Glucose oxidase coating density was adjusted to 1.5 U/ cm^2^, 3 U/ cm^2^, and 15 U/ cm^2^. The biomaterial with glucose oxidase to catalase ratio of 1:50 and glucose oxidase coating density of 1.5 U/cm^2^ has negligible hydrogen peroxide production and D-glucose consumption. Data are presented as mean ± SD (n = 8-10/group).

The working principle of the biomaterial indicates that it not only consumes oxygen but also consumes D-glucose and produces hydrogen peroxide and gluconic acid. First, hydrogen peroxide levels from different glucose oxidase and catalase combinations were measured. Biomaterials were coated on 96 well flat bottom plates and 100 μl of RPMI 1640 medium was incubated for 24 hours. Glucose oxidase density was fixed to 1.5 U/cm^2^ and the ratio of glucose oxidase to catalase was adjusted to 1:0, 1:1, 1:5, 1:10 and 1:50. As shown in Fig 2A, the hydrogen peroxide concentrations for control (no materials added) and glucose oxidase enzyme only group (glucose oxidase to catalase ratio is 1:0) were 0.86±0.11 μM and 402.83±16.77 μM, respectively. Without the presence of catalase, significant amount of hydrogen peroxide was accumulated in the media. With the presence of catalase, the hydrogen peroxide concentrations were decreased to 383.62±14.27 μM (1:1), 31.87±3.73 μM (1:5), 18.98±1.29 μM (1:10) and 0.87±0.13 μM (1:50), respectively. In order to avoid the influence of hydrogen peroxide, the ratio of glucose oxidase to catalase was fixed to 1:50 for D-glucose and different parameter measurements. D-glucose concentrations were measured after 4, 12, 24 and 48 hours incubation. D-glucose concentration kept decreased when the incubation time increased as shown in Fig 2B. With glucose oxidase coating density of 1.5 U/ cm^2^, 3 U/ cm^2^, and 15 U/ cm^2^, the D-glucose concentration was decreased to 89.99±0.26%; 88.05±0.76% and 69.98±1.35% respectively after 24 hours incubation. With 48 hours incubation, the D-glucose concentration was further decreased to 86.82±0.31%; 85.89±1.27% and 54.77±2.18% respectively. Many classical media are supplemented with approximately 5.5 mM D-glucose which approximates normal blood sugar levels *in vivo*. RPMI 1640 media contains 11.1 mM D-glucose. With 15% glucose consumption, the glucose level decreases from 11.1 mM to 9.4 mM which is still much higher than normal glucose level (5.5 mM). Hence, there was acceptable glucose consumption for the coating density of 1.5 U/ cm^2^ and 3 U/ cm^2^. For the optimized biomaterial, glucose oxidase density and glucose oxidase to catalase ratio were fixed to 1.5 U/cm^2^ and 1:50, respectively.

The production of gluconic acid may cause acidification of culture media. The pH of the cell culture media was measured after 48 hours incubation at 37°C in a humidified, 5% CO_2_ incubator with accurate pH meter (Fiber Optic pH Meter; World Precision Instruments). We found that the gluconic acid by-product accumulated in the medium did not affect the pH significantly (7.44±0.05 vs. 7.28±0.06, n = 4; control vs. with biomaterial). Most cell culture media contain a buffer system in their original formula to maintain the pH of the media at a certain range for a period of time. There are also supplemental buffer systems (i.e. HEPES, MOPS, etc.) available to minimize the pH changes for those pH sensitive cell lines. The RPMI 1640 Medium we used in this study contains a sodium bicarbonate buffer system (at 2.0 g/L). In addition, cell culture media is normally replaced by a fresh one twice a week. Therefore, the pH changes caused by the accumulation of gluconic acid will not be a factor for the cell growth in our hypoxia model.

### Generation of various oxygen tensions

**Fig 3:**
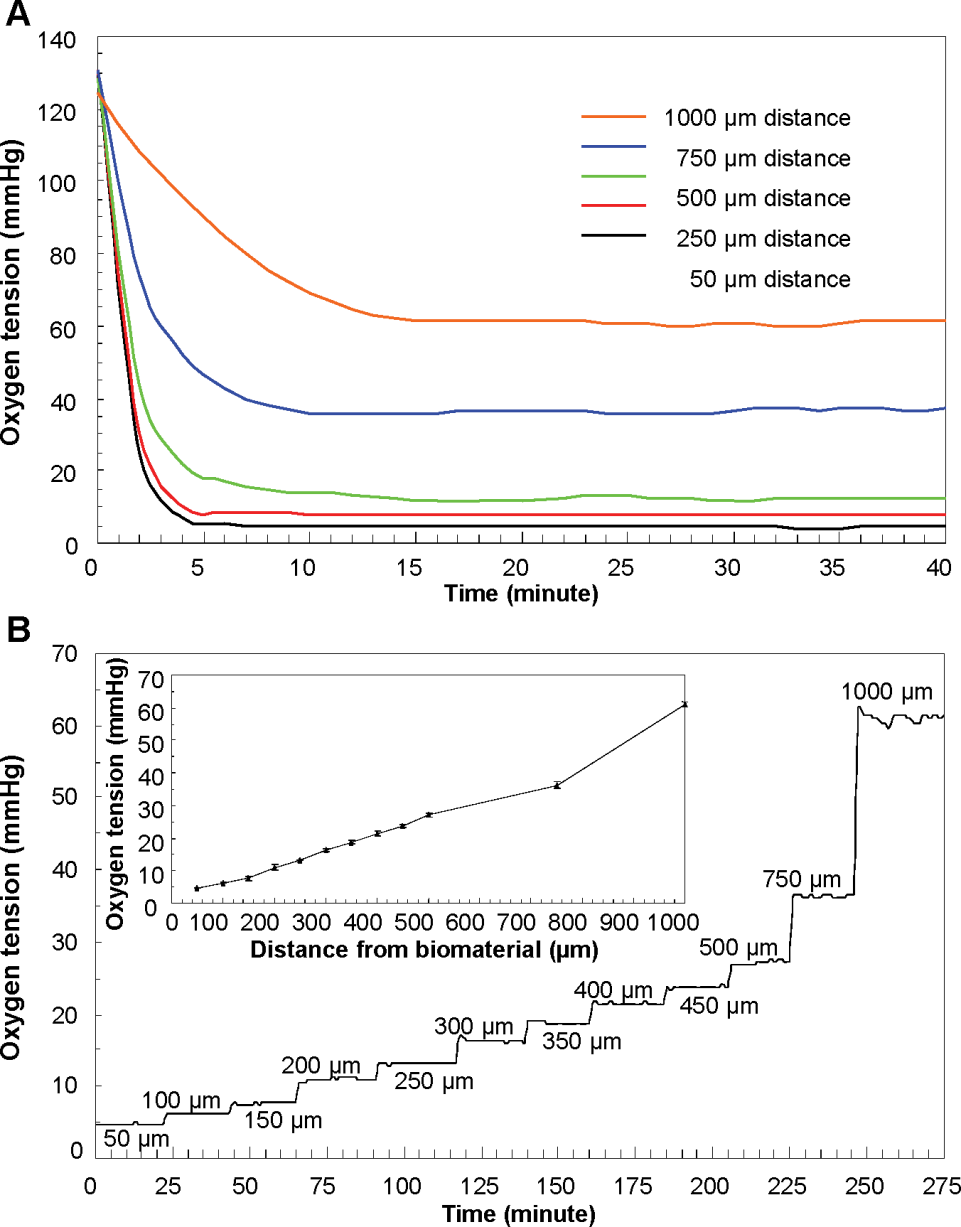
Oxygen tension characterization. (A) The response time to reach the steady state oxygen tension was measured at the difference distance from the biomaterial. (B) Z-axis oxygen tension in terms of various distances from the biomaterial. Inset figure provides the relationship between the distance from the biomaterial and generated oxygen tensions. Data are presented as mean ± SD (n = 12).

Biomaterials with glucose oxidase density of 1.5 U/cm^2^ and glucose oxidase to catalase ratio of 1:50 were coated on the bottom plate of the 3D-printed insert for 96-well plate. After aligning the oxygen microsensor to the bottom surface of the well plate, the insert was repositioned using a precision XYZ manipulator. 100 μl of RPMI 1640 medium containing 11.1 mM D-glucose was added. First, the response time to reach the steady state oxygen tension was measured at the difference distance from the biomaterial. The response time was hereby defined as the time required reaching 90% of the steady-state value. As shown in Fig 3A, it takes 3.8±0.3; 4.2±0.5; 5.2±0.1; 7.6±0.2 and 13.4±0.5 minutes to reach the steady oxygen tensions of 4.7±0.2; 13.2±0.6, 27.12±0.3; 36±2.4, 61.1±4.8 mmHg at distances of 50 μm, 250μm, 500 μm, 750μm, and 1000 μm, respectively. Oxygen tensions at different distances from the biomaterial were shown in Fig 3B. The initial data was recorded at 50 μm between the oxygen microsensor and biomaterial. After measuring the oxygen tension for 20 minutes, the insert with biomaterial was moved at increment of 50 μm. Oxygen tension increases as the distance from the biomaterial increases. The oxygen gradient was created in such a precise manner that every increment of 50 μm in the range of 0 to 500 μm in the Z-axis direction leads to 2.68±0.71 mmHg increase in the oxygen tension.

### Dynamic modulation of oxygen tensions

**Fig 4:**
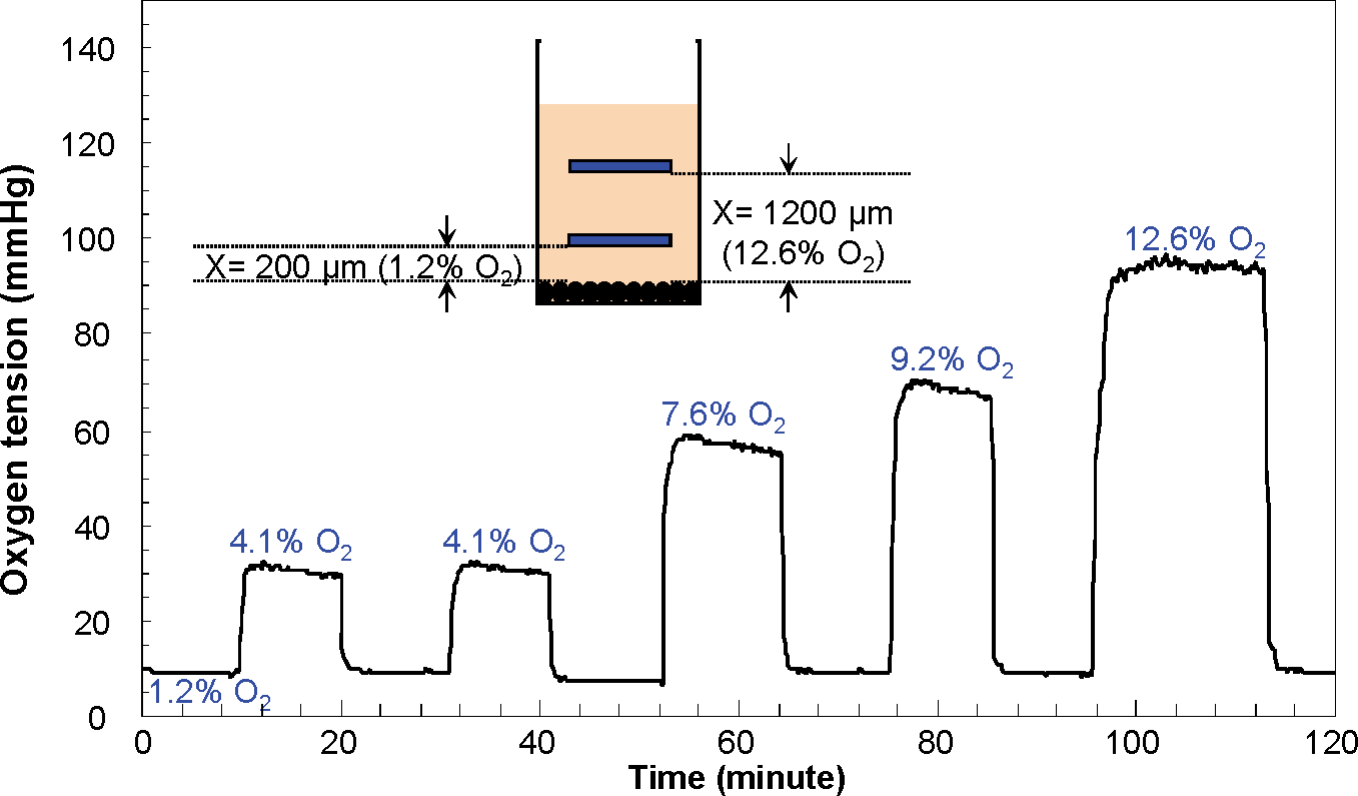
Dynamic modulation of oxygen tensions. The dynamic changes of oxygen tensions were evaluated by controlling the position of the biomaterial using a precision XYZ manipulator. Moving the inserts with biomaterials resulted in a rapid (<130 seconds) increase/decrease in oxygen tensions.

The dynamic changes of oxygen tensions were evaluated by modulating the distance from the biomaterial. Again, the oxygen microsensor was aligned to the bottom surface of the well plate and the insert with biomaterial was repositioned using a precision XYZ manipulator. As shown in Fig 4, moving the inserts with biomaterials resulted in a rapid increase/decrease in oxygen tensions (<130 seconds), whereas, after placing the inserts to the original position (1.2% O_2_ for this experiment), oxygen tensions were quickly recovered to almost baseline levels (<0.3% O_2_ deviation). 1.2% O_2_ level was generated when the distance between biomaterial and oxygen microsensor was 200 μm. The oxygen level was increased to 4.1%, 7.6%, 9.2% and 12.6% when the biomaterial was moved away from the oxygen microsensor 600 μm, 900 μm, 1000 um and 1200 μm distance, respectively.

### Cytotoxicity of the biomaterial

**Fig 5:**
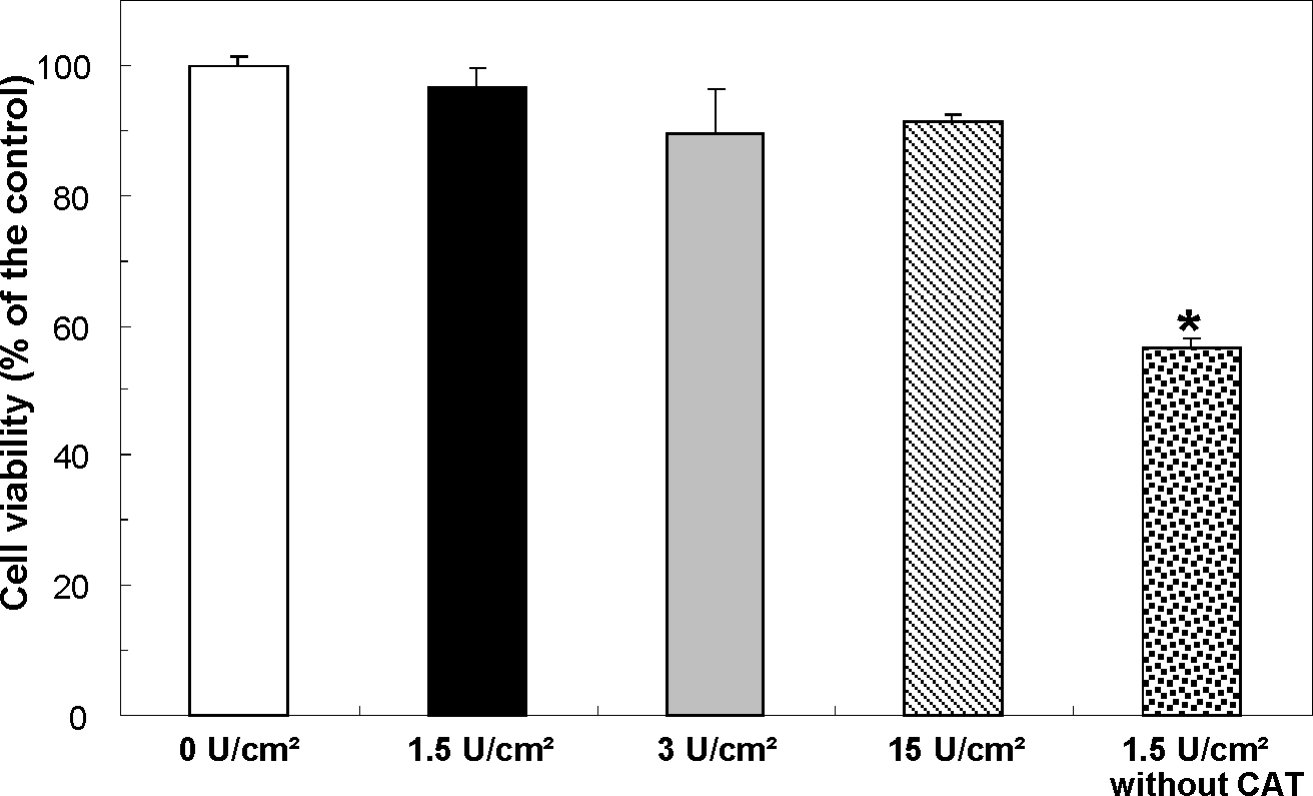
Cytotoxicity test. The biomaterial is biocompatible with different glucose oxidase enzyme densities from 0 to 15 U/cm^2^ (glucose oxidase to catalase ratio = 1:50), but toxic without catalase enzyme. Data are presented as mean ± SD (n = 12/group); *P<0.05 vs. the control group.

The biocompatibility of the biomaterials with different glucose oxidase enzyme densities was evaluated. RPMI 1640 medium was exposed to the biomaterials for 24 hours and the supernatant was collected. Rat macrophages (1 X 10^6^ cells/ml) were incubated in supernatant for another 24 hours. Fig 5 shows the cytotoxicity test results. Biomaterials with different concentrations of glucose oxidase showed biocompatible with enzyme densities ranging from 0 to 15 U/cm^2^. However, the biomaterial without catalase enzyme showed toxic. It indicates that the toxicity of biomaterials is mainly determined by the hydrogen peroxide levels and not other factors such as glucose, gluconic acid etc.

### Effect of hypoxia on the macrophages

Macrophages circulate within almost every tissue, they play a key role in phagocytosis and inflammation (by releasing several cytokines) to defend the host. In addition, macrophages also are capable of responding rapidly to hypoxia (low oxygen conditions) by altering their gene expression patterns. This way, macrophages are able to switch metabolism to anaerobic glycolysis, to produce more ATP and to increase their supply of oxygen and nutrients. It is well known that hypoxia and inflammatory signals share very similar transcriptional events in macrophages, including the activation of members of both the hypoxia-inducible factor (HIF-1α and HIF-2α) and nuclear factor κB (e.g., TNF-a) families [24]. We, therefore, examined hypoxia-induced changes in HIF-1α [25, 26] and TNF-α [27, 28] levels in macrophages to demonstrate that the developed biomaterials are able to effectively induce hypoxia at different levels.

**Fig 6:**
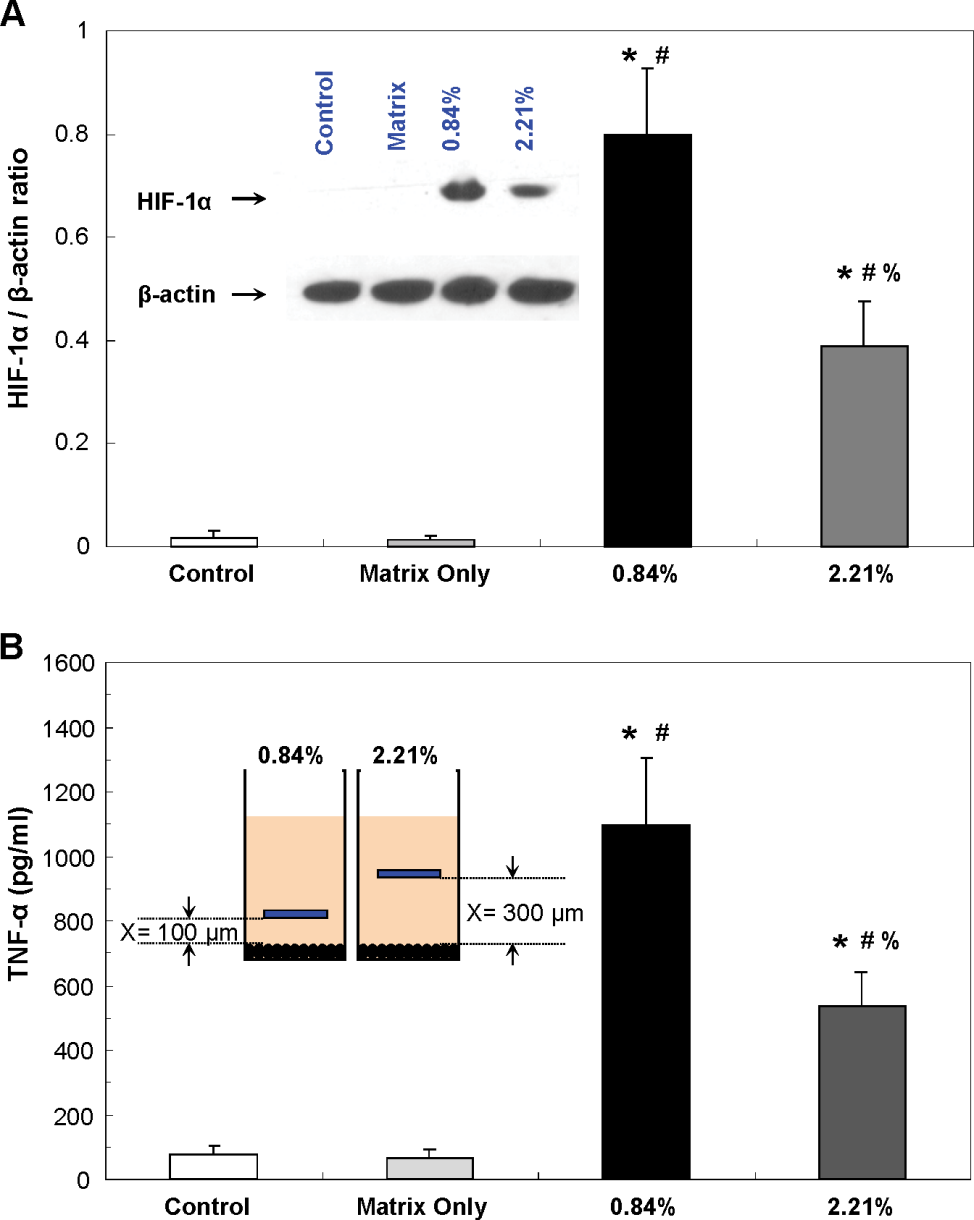
Effect of hypoxia on the peritoneal macrophages. (A) HIF-1α/β-actin ratios. Inset figure represents one of western blotting experiments. (B) TNF-α levels. Inset figure shows the distance between the biomaterial and cultured cells to generate two hypoxic levels, 0.84% and 2.21%. Data are presented as mean ± SD (n = 4-6/group); ^*^P<0.05 vs. the control group; ^#^P<0.05 vs. the matrix group; ^%^P<0.05 vs. “0.84% O_2_” group.

Primary peritoneal macrophages were cultured at 1 X 10^6^ cells/ml RPMI 1640 media in 24-well plates. Two hypoxic levels, 0.84% and 2.21%, were generated by placing two inserts with different pillar lengths (the distance between the biomaterials and cells of 100 μm and 300 μm, respectively) as shown in Fig 1C. After 24 hours incubation, both supernatant and cells from each well were collected. The levels of HIF-1α protein and TNF-α from hypoxia induced peritoneal macrophages were measured. Elevated HIF-1α activation and TNF-α expression in primary cultured peritoneal macrophages under hypoxia were detected as shown in Fig 6A and 6B. In addition, the macrophages cultured under more severe oxygen tension, 0.84% hypoxia, showed higher TNF-α levels and HIF-1α/β-actin ratios than the ones cultured under 2.21% hypoxia. The statistical analysis showed a significant difference between the normoxia (20.9% O_2_) and two different hypoxic conditions. Consequently, the experimental results demonstrate that oxygen tensions can be precisely controlled using the developed biomaterials.

## Discussion

Oxygen is critical in a wide variety of cellular signaling pathways. *In vivo* physiological oxygen tension ranges from 1 to 11%, which is well below ambient atmospheric oxygen level of 20.9% [29, 30]. Hypoxia is a major factor in the pathophysiology of many major diseases. To study these cellular responses, an *in vitro* cell culture system is needed in which the oxygen level can be maintained at a low level. In this study, a novel device has been developed to generate rapid, highly accurate and controllable oxygen tensions by manipulating the distance between the immobilized biomaterial and cultured cells through 3D-printed inserts. Our experimental results have shown that it took around 3.8 minutes to reach 0.5% hypoxia from normoxia (20.9%). Compared to the conventional hypoxic chambers / incubators which require more than 3 hours for the medium inside the cell culture plate to equilibrate to the gas outside of it [8], our proposed approach showed a ~47-fold faster response which allows researchers to mimic the sudden onset of ischemia in a stroke or heart attack.

Oxygen dissolves from air into the cell culture medium and oxygen consumption occurs on the surface of biomaterial. The oxygen equilibrium forms when oxygen consumption rate by biomaterial equals oxygen supply rate by air diffusion. By adjusting the distance between the cultured cells and biomaterial, various stable oxygen tensions can be achieved. The oxygen tension was controlled precisely with respect to the distance from the biomaterial, e.g., 2.68 mmHg per 50 μm increment in the range of 0 to 500 μm along the Z-axis. Our approach doesn’t require the cumbersome components of its forebears, such as syringe pumps or unwieldy interconnections. In addition, it can be reliably used into the cell incubators. 3D printing technology enables rapid, one-step fabrication of complex geometries not possible with planar lithography with high resolution [31, 32]. It aids to develop high precision oxygen tension generation inserts by controlling the pillar length.

Dynamic test results further showed that rapid (<130 seconds) and reversible (<0.3% O_2_ deviation) hypoxic conditions (from 12.6% to 1.2% oxygen tension) can be induced by changing the distance between the cells and biomaterials from 200 μm to 1200 μm. The results highlighted the potential of the developed hypoxia insert to be used for *in vitro* hypoxia / reoxygenation model of ischemia-reperfusion injury [33, 34].

In this study, we only used the biomaterial as an oxygen consuming material. However, the biomaterial also consumes the D-glucose in the cell culture media and generates hydrogen peroxide. All the chemical reactions happen on the biomaterial surface and diffuse into the media. It implies that hypoxic conditions with various D-glucose and hydrogen peroxide gradients can also be developed for different applications with different combinations of glucose oxidase and catalase enzymes [35–37]. The biomaterial can be further modified with oxygen storing enzymes such as myoglobin or nanoparticles such as cerium oxide nanoparticles to program the hypoxic conditions by eliminating saturation kinetics.

The developed biomaterial was applied to conventional cell culture microplates to control global oxygen of macro-environments as a proof of concept prototype. However, it can be applied to microfluidic devices to control local and complex oxygen of micro-environments that can closely mimic the microenvironment of living tissue [13]. The elegance of oxygen tension manipulation introduced by our new approach will drastically improve control and lower the technological barrier of entry for hypoxia studies. Further work adapting the new approach presented in this paper will allow a level of modularity and fidelity to the *in vivo* environment not yet seen in tissue culture studies. This biomaterial will serve as the basis for a new generation of experimental models previously impossible or very difficult to implement.

Taken together, we have developed a novel approach for on-demand generation of various oxygen tensions or gradients for *in vitro* hypoxia models. Compared to the current technologies, this approach allows enhanced spatiotemporal resolution and accuracy of the oxygen tensions. Additionally, it does not interfere with the testing environment while maintaining ease of use. This biomaterial can be applied for both conventional cell culture microplates and microfluidic devices to control global oxygen of macro-environments and local oxygen of micro-environments. Furthermore, advanced disease models can be developed by coating the biomaterial on the 3D-printed tissues or organs.
